# Predictors of early life milestones: Results from the Copenhagen Perinatal Cohort

**DOI:** 10.1186/s12887-019-1778-y

**Published:** 2019-11-09

**Authors:** Trine Flensborg-Madsen, Marie Grønkjær, Erik Lykke Mortensen

**Affiliations:** 1 Unit of Medical Psychology, Department of Public Health, University of Copenhagen, Øster Farimagsgade 5A, 1353 Copenhagen K, Denmark; 2 Center for Healthy Aging, University of Copenhagen, Blegdamsvej 3B, 2200 Copenhagen N, Denmark

**Keywords:** Developmental milestones, Family background, Pregnancy and delivery, Postnatal factors, Postnatal growth, Birth cohort, Longitudinal study

## Abstract

**Background:**

Pre- and postnatal factors have been found to be predictors of age at attaining milestones in infancy; however, the degree to which such factors are predictors of milestones in the subsequent years is less investigated. The aim was to conduct a systematic evaluation of a broad range of possible predictors of milestone attainment during the second and third years to identify factors that explain significant inter-individual variance.

**Methods:**

Mothers of 4009 children from the Copenhagen Perinatal Cohort (1959–61) were interviewed by a physician about 20 developmental milestones at a three-year examination. Milestones were related to: Language, Walking, Eating, Dressing, Social interaction, and Toilet training. Information on possible predictors was collected during pregnancy and at a 1- and 3-year follow-up.

**Results:**

Several pre- and postnatal factors were significantly associated with the timing of milestone attainment; especially parental social status, paternal age, sex, gestational age, birth weight, birth length, weight increase in the first year of life, and motor development during the first year of life. The significant predictors explained 16.2% of the variance in the Overall mean of milestones and 20.3% of the variance in milestones related to Walking. The most influential individual factor for the timing of milestone attainment was previous motor development during the first year of life. Additionally, sex was an important factor as girls were generally faster at attaining milestones. Parental social status was a consistent, but relatively week predictor.

**Conclusion:**

A notable amount of variance in the timing of milestones during the first three years of life can be explained by perinatal and early postnatal factors. The study provides evidence of developmental continuity as the main predictor of milestones in the second and third years was the speed of development during the first year.

## Background

During the course of the child’s first three years of life, notable developments occur in both fine and gross motor development as well as in language acquisition. Although age ranges for children’s attainment of milestones have been suggested, several studies have shown wide variations in the age of attainment of developmental milestones even in healthy children [1]. Even within the normal range, early attainment of several developmental milestones has been shown to be associated with beneficial outcomes later in life. Thus, previous research suggests that higher age at attainment of motor development is negatively associated with intelligence and cognitive function in both childhood and adulthood [2–8] as well as with higher neuroticism [9], anxiety and depression symptoms [10], and with psychiatric disorders, especially schizophrenia and alcohol use disorders [11–13]. Likewise, higher age at attainment of language milestones has been found to be predictive of lower intelligence in childhood [14, 15] as well as in adulthood [3, 16, 17] in addition to cognitive difficulties in general [8, 15, 18–20], attention and externalizing problems [21], and schizophrenia [22].

While the importance of developmental milestones is acknowledged for several outcomes, studies investigating potential predictors of the timing of these milestones are limited and mainly focus on language development. It has been found that especially socioeconomic status [23] and maternal education [24–26] is associated with language development, and additionally, girls have been found to generally attain language milestones faster than boys [24, 26]. However, early developmental factors such as gestational age [27–29] and low birth weight [30–32] have also been found to be associated with later attainment of language milestones. For postnatal factors, focus has especially been on parental factors, including the home environment [33–35] and emphasizes maternal responsiveness [36, 37] as an important predictor of language development. Finally, there have been studies of developmental continuity showing that the timing of earlier motor developmental milestones may be a good predictor of the attainment of language milestones [3, 38].

In a previous study, based on the Copenhagen Perinatal Cohort (CPC), a systematic evaluation of predictors of milestone attainment in the first year of life was conducted. This study found that predictors related to pregnancy and delivery such as birth weight and gestational age explained the largest proportion of variance in milestone attainment and that milestones attained during the first months of life to a larger extent could be explained by these factors than later milestones [39]. These findings raise questions concerning predictors of development during the second and third year of life because development during this period is substantially later than early perinatal predictors and because of the increasingly complex behaviour during this period. Thus, behavioural milestones achieved during the first year of life primarily reflect motor development while later milestones increasingly reflect cognitive and language development. This change could lead to changes in the important predictors—for example, parental social status was found to be a relatively weak and inconsistent predictor of milestones during the first year of life while this variable is expected to be associated with the child’s cognitive and language development. Similarly, sex differences would be expected with regards to language development, while a previous study of motor development during the first year observed no sex differences [23].

While the vast majority of studies on predictors of milestone development focused specifically on language development, no studies have compared prediction of other developmental milestones that appear at the same age as language milestones, such as for example milestones related to gross and fine motor development, eating skills, toilet skills and social interaction. Hence, the investigated predictors represent only a small fraction of possible predictors hypothesized to be associated with behavioural development and the timing of milestones, leaving many potential predictors unexplored.

The primary objective of the study was to conduct a systematic evaluation of a broad range of possible predictors of milestone attainment during the second and the third years of life. While a previous study found factors related to pregnancy and delivery to be the most important predictors [39], we expected factors related to family background and postnatal factors to have stronger influence on development during the second and third years of life.

## Methods

### Study sample

The study sample comprises members from the Copenhagen Perinatal Cohort (CPC) which consists of 9125 children born to 8949 mothers at the Copenhagen University Hospital between 1959 and 1961. Admittance was based on area of residence (Copenhagen), but some were referred due to obstetrical complications or single mother status. When the cohort was established, demographic, socioeconomic, prenatal, and postnatal data were recorded prospectively during pregnancy, at delivery, at a 1-year follow-up, and at a 3-year follow-up. Detailed descriptions of the data collection can be found elsewhere [40]. A total of 8395 infants survived the first month after birth and of these, information on at least one developmental milestone was available for 4120 individuals, but 111 multiples were excluded since data for twins and triplets are not statistically independent. The final sample thus included 4009 singletons (2047 boys and 1962 girls).

### Developmental milestones

At the 3-year follow-up assessment, the mothers were interviewed and asked to retrospectively recall the child’s age at attaining 20 developmental milestones. Descriptive information on the 20 developmental milestones is shown in Table 1. The rate of missing data on each milestone varied from 4.8% (walking) to 49.0% (turning head in the right direction). To be able to conduct a principal component analysis of all 20 milestones, the expectation–maximization (EM) algorithm was used to construct a dataset with missing milestone data replaced by imputed data. The first six components explained 71% of the variance, and both varimax and promax rotation defined the following six factors: Language (milestones 1–6), Walking (milestones 7–8), Eating (milestones 9–10), Dressing (milestones 11–12), Social interaction (milestones 13–17), and Toilet training (milestones 18–20); for details see Sørensen et al. [13]. In addition, an Overall mean of scores on all 20 milestones was calculated. All milestone mean scores were derived by linearly standardizing each milestone to a mean of 0 and a standard deviation of 1, and by calculating the means of the standardized scores. Finally, the six factor means and the Overall mean were re-standardized to a mean of 0 and a standard deviation of 1. Data could be missing for one or more milestones included in a milestone mean, and in these cases the mean of the available milestone scores was calculated. If scores on all milestones included in a mean were missing, the corresponding mean was scored as missing. Since the study sample only included individuals with data available for at least one milestone, there were no missing data on the Overall mean of milestones..

**Table 1:** Developmental milestones (specified in months) from the Copenhagen Perinatal Cohort

Composite milestone means:	Developmental milestones	Description	*N*	Mean (SD)	Range
LANGUAGE	1. ‘Turning head in the right direction’	The child can turn his/her head in the right “direction” if you e.g. say: where is “mom”, “dad”, “the light” etc.	2045	12.3 (4.6)	3–30
	2. ‘Naming objects/animals’	The child can name a few, familiar objects or animals with their true names	2695	18.1 (5.5)	9–34
	3. ‘Naming objects/animals in pictures’	The child can name a few, familiar objects or animals in pictures with their true names	2662	20.0 (5.6)	10–36
	4. ‘Forming a sentence’	The child can put at least three words together to form a “sentence”	2827	22.9 (5.2)	12–36
	5. ‘Speaking properly’	The child can speak properly	2201	28.6 (5.1)	16–36
	6. ‘Sharing experiences’	The child can talk about what it has experienced	2892	28.9 (4.6)	18–36
WALKING	7. ‘Walking’	The child can walk around unassisted in the living room	3815	13.8 (2.7)	9–24
	8. ‘Climbing stairs’	The child can climb stairs unassisted	3323	21.1 (5.3)	11–36
EATING	9. ‘Drinking from cup’	The child can drink from a cup without assistance	3370	17.5 (4.6)	7–30
	10. ‘Eating with spoon’	The child can eat with a spoon without assistance	3407	20.1 (5.0)	10–36
DRESSING	11. ‘Putting on socks’	The child can put socks on by itself	2577	28.9 (5.4)	14–36
	12. ‘Doing buttons’	The child can do buttons	2536	29.9 (5.0)	18–36
SOCIAL INTERACTION	13. ‘Building tower’	The child can build a tower of 4–5 ordinary rectangular blocks	2575	20.5 (5.8)	10–36
	14. ‘Helping at home’	The child shows interest in helping at home by imitating parents (e.g. by laying the table or other domestic tasks)	3446	24.5 (5.6)	13–36
	15. ‘Picking up things’	The child can pick up things in the apartment if requested	3304	22.6 (5.7)	12–36
	16. ‘Playing with peers’	The child plays with children of the same age (e.g. rolling a ball to each other)	2866	20.8 (5.8)	10–36
	17. ‘Distinguishing boys and girls’	The child can distinguish between boys and girls	2283	27.0 (5.5)	15–36
TOILET TRAINING	18. ‘Bowel control’	The child can tell when it needs to defecate	3493	24.9 (6.0)	12–36
	19. ‘Dry during the day’	The child is dry during the day and tells when it needs to go to the toilet	3514	25.6 (5.9)	12–36
	20. ‘Dry during the night’	The child is dry during the night	2963	26.2 (6.3)	12–36

### Predictor variables

For comparison reasons, we primarily included variables that were also included in the previous study of predictors of 1-year milestones [39] and added variables describing growth and development during the second and third years. We also used the same categorization of potential predictor variables in four domains. Descriptive information on each potential predictor variable is found in Table 2.

**Table 2:** Potential predictors from the Copenhagen Perinatal Cohort and associations with the Overall mean of milestones

	*N*	Mean (SD)	Range	r^a^
FAMILY BACKGROUND				
Parental social status (1 to 8 point scale)	3421	4.5 (1.8)	1–8	−0.04
Parity (first child, Yes %)	4001	48.9%	First/not first	0.02
Maternal age	3988	26.6 (6.5)	14–48	0.003
Paternal age	3886	30.2 (7.6)	16–71	0.02
Single mother, prenatally (yes %)	3998	23.2%	Yes/No	−0.002
PREGNANCY AND DELIVERY				
Sex (female %)	4009	48.9%	Female/male	−0.18***
Gestational age (weeks)	3340	39.1 (2.4)	27–46.5	−0.11***
Birth weight (grams)	3992	3247 (586)	850–5450	−0.09***
Birth length (cm)	3989	51.0 (2.7)	24–60	−0.07***
Head circumference at birth (cm)	3734	34.5 (1.7)	25.1–39.7	−0.07***
BMI at birth	3988	12.4 (1.4)	7.2–17.5	−0.09***
Mother’s smoking in last trimester (yes %)	3954	45.8%	Yes/no	0.02
Mother’s attitude towards pregnancy (unwanted %)	3894	45.1%	Wanted/unwanted	0.03
POSTNATAL FACTORS				
Breastfeeding (months)	3432	3.4 (2.8)	0.3–12	− 0.06***
Mother’s employment (not employed at 1-year)	2750	88.9%	Yes/no	0.02
Lived in full-time institution at some point in first year (yes %)	3945	7.3%	Yes/no	0.04*
Daycare institution at some point in the first year (yes %)	3932	10.7%	Yes/no	−0.0004
POSTNATAL GROWTH AND DEVELOPMENT				
Weight increase, first year (kg)	3425	7.1 (1.3)	3–20.6	0.00
Length increase, first year (cm)	3374	25.1 (3.6)	14–46.5	0.05**
Head increase, first year (cm)	3193	12.8 (1.7)	5.5–21.3	0.08***
BMI increase, first year	3359	5.5 (2.0)	−2.7-22.4	0.02
Weight increase, age 1–3	2849	4.5 (1.4)	−4.1-11.4	0.004
Length increase, age 1–3	2871	20.4 (3.7)	0–40	−0.03
Head increase, age 1–3	2880	3.4 (1.2)	0–13.5	0.004
Overall mean of 1-year milestones	4008	0.05 (0.7)	−2.17-2.99	0.34***

^a^ Pearson correlations were used to evaluate bivariate associations between each predictor and Overall mean of milestones (point-biserial correlation for binary predictors)

^*^p < 0.05; ^**^ *p* < 0.01; *** *p* < 0.001

#### Family background

*Parental social status* was based on the social grouping of the Centre International de l’Enfance [41] and was assessed at the 1-year examination on a 1 to 8 point scale (with 8 being the highest). The score is based on a combination of the following four factors: A) the occupation of the breadwinner; B) the way in which the breadwinner earns his/her wages (public relief, daily wage, weekly wage, monthly salary and own business or capital); C) the education of the breadwinner; and D) the character of the living accommodation (its size, the number of persons per room, its position, etc.) [42]. *Parity*, *maternal age*, *paternal age,* and *single mother status prenatally* were obtained from interviews by physician, A. L. Villumsen [43] who interviewed all the women.

#### Pregnancy and delivery

The following variables were obtained from the hospital records: *Sex of the child*, *gestational age*, *birth weight*, *birth length*, and *head circumference at birth*. *Mother’s smoking in last trimester* and her *attitude towards the pregnancy* were obtained from interviews with the mother.

#### Postnatal factors

Information on *breastfeeding*, *mother’s employment*, *institutionalization* of the child, and *daycare institution* during the first year were obtained from the 1-year follow-up examination of the child.

#### Postnatal growth and development

*Weight*, *length*, and *head increase during the first year of life* were derived from the delivery and the 1-year follow-up measures. *Weight*, *length*, and *head increase from age one to three* were calculated from the 1-year and the 3-year follow-up measures. Finally, the overall mean of the 1-year milestones analyzed previously [39] was included as a predictor to investigate developmental continuity between the first year and the following two years. The 12 motor milestones included in the Overall 1-year mean are described in detail elsewhere [2, 39].

### Data analysis

To evaluate bivariate associations between each potential predictor and the Overall mean of milestones, Pearson correlations were used. The rate of missing data on each potential predictor varied from 0% (sex) to 31.4% (mother’s employment), and the rate of missing data on the milestone outcome means varied from 0% (Overall milestone mean) to 25% (Dressing mean). Due to missing data, all subsequent analyses were conducted using Full Information Maximum Likelihood (FIML) analyses [44]. In the FIML analyses, we used structural equation modelling facilities of Stata 14 (College Station, TX: StataCorp LP, USA) to use all available information in each particular analysis. First, multiple linear regression models were conducted for each domain of predictors separately (‘family background’, ‘pregnancy and delivery’, ‘postnatal factors’, and ‘postnatal growth’) with each of the seven milestone means as the outcome. In these analyses, all variables in each domain were included in the same model (Table 3). BMI was not included in these regression models to avoid excessive co-linearity of BMI with other covariates related to size. Second, a full regression model was conducted for each milestone mean in which variables with a *p* value of 0.10 or below in the domain-specific analyses were included (Table 4). An overview of the variance explained by each of the four domains and the full model is shown in Table 5.

**Table 3:** Linear regression analyses of predictor variables and milestone means within predictor domains

	Overall mean of milestones	Language	Walking	Eating	Dressing	Social interaction	Toilet training
	*β*	*β*	*β*	*β*	*β*	*β*	*β*
FAMILY BACKGROUND	(R-sq = 0.003)	(R-sq = 0.011)	(R-sq = 0.007)	(R-sq = 0.005)	(R-sq = 0.005)	(R-sq = 0.006)	(R-sq = 0.003)
Parental social status (1–8)	− 0.05**	− 0.10***	− 0.05**	− 0.04	0.03	− 0.07***	0.06**
Parity (first born)	−0.02	− 0.05*	− 0.03	0.03	0.05*	− 0.02	− 0.01
Maternal age	− 0.01	0.02	− 0.01	− 0.02	0.04	− 0.03	− 0.02
Paternal age	0.03	− 0.03	0.07**	0.09***	−0.05	0.04	0.01
Single mother, prenatally	0.001	−0.02	−0.003	0.02	−0.01	− 0.01	0.01
PREGNANCY AND DELIVERY	(R-sq = 0.052)	(R-sq = 0.036)	(R-sq = 0.034)	(R-sq = 0.010)	(R-sq = 0.089)	(R-sq = 0.013)	(R-sq = 0.062)
Sex (female)	−0.19***	−0.17***	− 0.01	− 0.000	− 0.26***	−0.06***	− 0.23***
Gestational age	−0.04*	0.01	−0.05*	− 0.04	−0.08***	0.01	−0.04
Birth weight	−0.08*	−0.04	− 0.12***	−0.12**	− 0.12**	−0.05	− 0.04
Birth weight squared	0.05*	0.03	0.05*	0.05	0.01	0.04	0.04
Birth length	0.04	0.01	−0.01	0.10**	0.19***	0.02	0.02
Head circumference at birth	−0.02	−0.05	0.02	−0.02	0.03	−0.04	− 0.03
Mother smoked in last trimester	0.01	−0.001	0.01	−0.002	0.003	0.02	0.02
Mother’s attitude towards pregnancy (unwanted)	0.03	0.04*	0.01	−0.01	− 0.04*	0.06***	− 0.04*
POSTNATAL FACTORS	(R-sq = 0.005)	(R-sq = 0.004)	(R-sq = 0.006)	(R-sq = 0.004)	(R-sq = 0.003)	(R-sq = 0.005)	(R-sq = 0.001)
Breastfeeding	−0.06***	− 0.05**	− 0.04*	− 0.04*	−0.005	− 0.04*	− 0.02
Mother’s employment (not employed at 1-year)	0.03	−0.01	− 0.04	0.02	0.06*	0.05*	0.02
Full-time institution at some point in the first year	0.02	0.02	0.04**	0.03	0.01	0.03	0.01
Daycare institution at some point in the first year	−0.02	0.03	0.02	−0.04	− 0.02	− 0.02	− 0.03
POSTNATAL GROWTH AND DEVELOPMENT	(R-sq = 0.116)	(R-sq = 0.045)	(R-sq = 0.198)	(R-sq = 0.048)	(R-sq = 0.030)	(R-sq = 0.055)	(R-sq = 0.040)
Weight increase, first year	−0.01	− 0.01	− 0.04*	− 0.03	0.08**	− 0.04	− 0.00
Length increase, first year	−0.03	− 0.03	− 0.06*	0.03	− 0.05	−0.01	− 0.02
Head increase, first year	0.02	0.04	0.05*	−0.01	0.08**	−0.02	0.06*
Weight increase, age 1-3	0.03	0.02	0.01	0.00	0.01	0.02	−0.00
Length increase, age 1-3	−0.06*	− 0.04	− 0.08**	0.00	0.02	− 0.01	− 0.04
Head increase, age 1-3	0.01	0.00	0.02	0.01	0.01	−0.00	0.03
Overall mean of 1-year milestones	0.34***	0.20***	0.44***	0.22***	0.12***	0.24***	0.18***

^*^p ≤ 0.05; ^**^ p ≤ 0.01; *** p ≤ 0.001

**Table 4:** Linear regression analysis of selected predictor variables and milestone means (models include all listed variables)

	Overall mean of milestones	Language	Walking	Eating	Dressing	Social interaction	Toilet training
	*β*	*β*	*β*	*β*	*β*	*β*	*β*
FAMILY BACKGROUND							
Parental social status (1–8)	−0.04*	− 0.08***	− 0.04*	− 0.04*	0.02	− 0.05**	0.05*
Parity (first born)	0.02	−0.03	0.02	0.06**	0.06**	0.00	0.01
Paternal age	0.01	−0.02	0.05**	0.06**	−0.03	0.01	−0.01
PREGNANCY AND DELIVERY							
Sex (female)	−0.22***	−0.18***	− 0.03	−0.01	− 0.27***	−0.08***	− 0.24***
Gestational age	−0.00	0.04	0.02	−0.02	−0.05*	0.04	−0.01
Birth weight	−0.07*	−0.05	− 0.05	−0.11**	− 0.06	−0.06	− 0.05
Birth weight squared	0.01	0.02	0.01	0.00	0.02	0.01	0.01
Birth length	0.05	0.02	−0.01	0.13***	0.11**	0.04	0.02
Mother’s attitude towards pregnancy (unwanted)	0.00	0.01	−0.01	−0.02	− 0.04*	0.04*	− 0.03
POSTNATAL FACTORS							
Breastfeeding	−0.01	−0.01	0.01	−0.02	0.01	−0.01	− 0.01
Mother’s employment (not employed at 1-year)	0.04	0.01	−0.02	0.01	0.05	0.05*	0.03
Full-time institution at some point in the first year	−0.01	− 0.00	0.01	0.01	0.02	0.00	0.01
Daycare institution at some point in the first year	−0.04*	0.00	0.02	−0.04	−0.04	− 0.04	−0.03
POSTNATAL GROWTH AND DEVELOPMENT							
Weight increase, first year	−0.07**	−0.06*	− 0.04*	−0.06*	− 0.02	−0.06*	− 0.05*
Length increase, first year	0.01	−0.00	−0.07*	0.06*	0.01	0.03	0.00
Head increase, first year	−0.03	0.01	0.02	−0.03	0.03	−0.03	−0.01
Length increase, age 1–3	0.00	0.02	−0.07**	0.01	0.06*	0.02	−0.02
Overall mean of 1-year milestones	0.35***	0.21***	0.43***	0.22***	0.15***	0.24***	0.19***

^*^p < 0.05; ^**^ *p* < 0.01; *** *p* < 0.001

**Table 5:** Variance in milestone means explained (R^2^) by models of predictors

	R^2^						
Statistical model:	Overall mean of milestones	Language	Walking	Eating	Dressing	Social interaction	Toilet training
Family background	0.003	0.011	0.007	0.005	0.005	0.006	0.003
Pregnancy and delivery	0.052	0.036	0.034	0.010	0.089	0.013	0.062
Postnatal factors	0.005	0.004	0.006	0.004	0.003	0.005	0.001
Postnatal growth and development	0.116	0.045	0.198	0.048	0.030	0.055	0.040
Final model	0.162	0.084	0.203	0.059	0.118	0.069	0.097

Preliminary analyses showed that birth weight was quadratically associated with the Overall mean of milestones in domain-specific analyses, and consequently quadratic terms were included for this variable. All statistical analyses were conducted using *Stata Statistical Software: Release 14* (College Station, TX: StataCorp LP, USA).

## Results

The study sample characteristics in Table 2 show that the sample consisted of 51.1% boys and that the mean birth weight was 3247 g, with a mean weight increase in the first year of 7.1 kg. Almost half of the mothers (45.8%) smoked in the last trimester and the majority of the mothers (88.9%) were not employed when the child was one year of age.

Throughout this study, positive associations imply higher age (slower attainment of milestones) with larger values of the predictor, while negative associations imply faster attainment with larger values of the predictor.

### Bivariate correlations

Several potential predictors from the four domains were associated with the Overall mean of milestones in bivariate analyses (Table 2). The strongest correlation (r = 0.34) was observed between the 1-year and 3-year Overall milestone means, and in addition especially variables from the domain ‘pregnancy and delivery’ were significantly associated with the Overall 3-year mean of milestones. Thus, being a girl (r = − 0.18), having a higher gestational age (r = − 0.11), a larger birth weight (r = − 0.09), a larger birth length (r = − 0.07), a larger head circumference at birth (r = − 0.07) and a larger BMI at birth (r = − 0.09) were all significantly associated with faster attainment of milestones in general. Furthermore, breastfeeding (r = − 0.06) and stay at a full-time institution during the first year (r = 0.04) were significant predictors from the domain of ‘postnatal factors’, while length and head increase (r = 0.05 and 0.08) in the first year were significant predictors from the domain ‘postnatal growth’. None of the predictors in the domain of ‘family background’ were significantly associated with the Overall mean of milestones.

### Predictor domains

Analysing domains of predictors separately in four different models for each of the seven means, the patterns from the bivariate analyses were repeated (Table 3). Thus, variables from the domain ‘pregnancy and delivery’ were significantly associated with the seven means; especially sex, gestational age, birth weight, and birth length were found to be associated with later milestone attainment. However, it should be noted that there were no sex differences with regard to Walking and Eating, suggesting that boys and girls are developing similarly in these areas. In the domain of ‘family background’, especially parental social status was a significant predictor (Overall mean: β = − 0.05; *p* ≤ 0.01), implying faster attainment of milestones with higher social status. Similarly, breastfeeding was a significant variable from the domain of ‘postnatal factors’ (Overall mean: β = − 0.06; *p* ≤ 0.001), implying faster attainment of milestones with longer duration of breastfeeding. ‘Postnatal growth’ in the first year, especially head increase, was significantly associated with several milestone means with estimates implying that a large increase in head circumference was associated with later attainment of milestones. In contrast, large weight and height increase was associated with faster attainment of milestones except for an association between weight increase and later attainment of Dressing milestones. However, clearly the strongest predictor was the Overall mean of 1-year milestones with highly significant coefficients for all 3-year milestone means and particular strong associations with the Overall 3-year mean and attainment of Walking milestones.

### Model of selected predictor variables

The model with selected predictors included variables from the multiple regression models conducted for each domain of predictors (Table 3) with a *p* value of ≤0.10 for at least one of the seven means. All estimates shown in Table 4 are adjusted for the other predictor variables in the table. In the domain ‘family background’, higher parental social status was associated with significantly faster attainment of all milestone means except for a lack of an association with Dressing and significantly later attainment of milestones related to Toilet training. Being the firstborn was associated with later attainment of milestones related to Eating and Dressing while higher paternal age was associated with later attainment of milestones related to Walking and Eating.

The strongest pre- and perinatal associations with milestones were found in the domain ‘pregnancy and delivery’, where especially female sex was associated with faster attainment of all milestone means except milestones related to Walking and Eating. Gestational age was only significantly associated with faster attainment of milestones related to Dressing, while birth weight was related to the Overall mean and faster attainment of Eating milestones and birth length was significantly associated with later attainment of milestones related to Eating and Dressing. The mother’s attitude towards pregnancy (being unwanted) was associated with faster attainment of milestones related to Dressing and later attainment of milestones related to Social interaction.

Most predictors in the domain ‘postnatal factors’ were not associated with any milestone means, but mother’s unemployment was significantly associated with later attainment of milestones related to Social interaction, and spending time in a day care institution was associated with faster development as reflected in the Overall milestone mean.

In the domain ‘postnatal growth’, weight increase in the first year of life was associated with faster development, as reflected in significant associations with all milestone means except milestones related to Dressing. Length increase in the first year was significantly associated with faster attainment of milestones related to Walking and later attainment of milestones related to Eating. Length increase during the second and third year was also significantly related to faster Walking, but also to later attainment of milestones related to Dressing. However, in this full model the coefficients for the Overall mean of 1-year milestones were only slightly reduced or actually increased, and thus speed of development during the first year of life remained the main predictor of speed of development during the subsequent two years. The strongest associations were seen for the Overall 3-year milestone mean and for Walking while the weakest association was observed for Dressing.

### Explained variance

The final model explained 16.2% of the variance in the Overall mean of milestones (Table 5). Among the seven milestone means, the largest variance explained by the final model was for the milestone mean Walking (20.3%), while the smallest variance explained by the final model was for the milestone mean Eating (5.9%). The domain ‘postnatal growth and development’ contributed most of the explained variance for all milestone means except for the Dressing and Toilet Training means where the ‘pregnancy and delivery’ domain explained 8.9 and 6.2% of the variance because of the large sex differences in milestones related to these domains. In fact, sex difference seemed to contribute substantially to making the ‘pregnancy and delivery’ domain the second largest contributor to explained variance apart from the ‘postnatal growth and development’ predictors that directly describe the child’s growth and behavioural development.

## Discussion

The aim of the study was to conduct a systematic evaluation of a range of possible predictors of milestone attainment during the second and third years of life. We were able to explain 16.2% of the variance in the Overall mean of milestones with a model summarizing the findings in the four predictor domains ‘family background’, ‘pregnancy and delivery’, ‘postnatal factors’, and ‘postnatal growth and development’. This is close to the 18.5% explained variance in a previous study of milestones attained during the first year of life [39], but remarkably, only 6.3% of the variance in 3-year milestones was explained when the Overall 1-year milestone mean was not included in the final model. This suggests that the influence of early life factors such as family background, factors related to pregnancy and delivery and postnatal influences on the child’s behavioural development gradually becomes weaker as the child is influenced by environmental factors and develops more complex cognitive, linguistic and social behaviour. In contrast, growth and in particular motor development during the first year becomes a crucial predictor of age of attaining milestones during the second and third year of life. Thus, the present study provides evidence of developmental continuity and corroborates studies showing associations between age of attaining motor developmental milestones and later language milestones [3, 38].

From the domain ‘family background’, higher parental social status was found to be associated with faster attainment of milestones related to Language, Walking, Eating, and Social interaction. Parental social status is in this study defined as a joint measure of both educational and occupational aspects as well as the character of the living accommodation, and the results are therefore in accordance with previous studies showing that socioeconomic status [23] and maternal education [24–26] are positively associated with language development, in particular since we observed the strongest association for milestones related to Language. Cognitive and language development are closely related, and in the Copenhagen Perinatal Cohort (CPC), language milestones predict young adult and midlife intelligence [16, 45]. Since the CPC measure of parental social status is a strong predictor of offspring adult intelligence [46], a relatively strong association between parental social status and Language milestones was expected, but the strength of the association was considerably weaker than the association between sex and Language milestones.

Other studies support our significant finding of faster language development in the firstborn child [24, 47]. Research on the relationship between father involvement and outcomes in the child has found that fathers may influence various aspects of development, including executive functioning [48, 49]. In this study we found that children of older fathers were significantly later at attaining milestones related to Walking and Eating which may corroborate the previous studies if it is assumed that fathers engage less in the child’s development with increasing age. The association between higher paternal age and later motor development was also observed for all 1-year milestones in a previous study [39].

A previous study found that milestone attainment during the first year was primarily predicted by factors related to pregnancy and delivery, and the present findings show that these factors are also important predictors of milestones attained during the second and third year. However, milestones during the first year were predicted by several foetal growth factors such as gestational age, birth weight and head circumference while the primary predictor in the present study was the child’s sex. The present study thus replicated findings showing that girls are significantly faster than boys at attaining milestones in general, and especially those related to language [24, 26], toilet training [50] and in our study also milestones related to dressing and social interaction. Thus, the fact that no sex differences were observed with respect to milestones related to Walking and Eating is a remarkable finding. No sex differences were identified in the previous study of motor milestones attained during the first year [39], and the pattern of results suggests that sex differences appear as the child develops more complex abilities and behaviour.

The effects of birth weight and length were somewhat diluted in the final model compared to the domain analyses, but a previous study showed that birth weight was an important predictor of 1-year milestones [39], and thus effects of birth weight may be partly mediated by the Overall 1-year milestone mean, an assumption supported by the fact that birth weight was significantly associated with all milestones in a model without the Overall 1-year milestones mean as a predictor. Thus, the findings may be in accordance with previous studies showing that low birth weight is a significant predictor of slow milestone attainment, especially those related to language development [30–32]. A large birth length was in this study associated with slower attainment of milestones related to Eating and Dressing; this may be explained by poorer fine motor skills with increasing limb length in the first years. The mother’s attitude to the pregnancy was not significant in our previous study [39], and it is difficult to interpret the inconsistent findings for milestones related to Dressing and Social interaction.

In this study we did not have variables to describe the level of stimulation and parental responsiveness which in previous studies have been found to be particularly important for language development [33, 36, 37]. This may explain why only few variables from the domain ‘postnatal factors’ were predictors of milestone attainment. However, our results show that the mother’s unemployment in the first year of life is associated with later attainment of milestones related to Social interaction and in the domain analyses also milestones related to Dressing. Full-time institutional care was only associated with later attainment of milestones related to Walking in the domain analyses while time in day-care institution was associated with the Overall mean in the final models. In the domain analyses, breastfeeding was associated with the Overall mean and the milestone means Language, Walking, Eating and Social interaction, but all associations became non-significant in the final models. Breastfeeding has previously been shown to be associated with faster attainment of motor milestones during the first year in the CPC [39], and therefore the association with milestones attained during the second and third years may be partly mediated through physical growth and the Overall 1-year milestone mean.

Concerning ‘postnatal growth’, weight increase in the first year was by far the most important predictor of milestone attainment in general. Thus, except for the Dressing mean, a large weight increase in the first year was associated with faster attainment of milestones in all areas. Length increase in both the first and the subsequent years was associated with faster attainment of milestones related to Walking while length increase in the first year was related to slower attainment of milestones related to Eating, and length increase in the subsequent years to slower attainment of milestones related to Dressing. The latter is in accordance with the results showing that birth length is associated with slower attainment of milestones related to both Eating and Dressing and underlines that motor skills related to getting dressed is more difficult with long extremities. A previous study based on the CPC found that weight increase in the first year was associated with faster attainment of all motor-developmental domains in the first year of life [2]. We were, however, not able to locate studies that investigated the effects of physical growth on milestone attainment in the subsequent years after the age of one.

Although the current study sample primarily consisted of essentially healthy infants, the study sample is not representative of the Danish population at the time when the cohort was established. This is because the maternity departments at the Copenhagen University Hospital primarily accepted pregnant women with previous pregnancy complications, pregnant women expected to undergo complicated delivery, and single mothers for whom delivery at home would be inconvenient (at the time of investigation it was a standard procedure to give birth at home) [42]. It is also an open question whether the specific results can be generalized to younger birth cohorts, although we expect that major predictors such as parental social status, sex, and measures of development during the first year of life would also be significant in younger generations. A possible limitation of our study is that milestones related to Walking may be attained in both the first and second year of life, and this may explain the high correlation between the Overall 1-year milestone mean and the 3-year milestones related to Walking. It is also a limitation that some of our variables were binary codings of complex phenomena such as mother’s employment and the child’s stay in full-time and day-care institutions.

The population-based and longitudinal design of the study also provides a number of strengths. Although information on milestones was retrospectively recalled by the mother at a 3-year follow-up examination of the child, information on predictors was registered prospectively and before this examination. Previous studies have not included as wide a range of possible predictors and milestones from several behavioural domains. However, comparison with a previous study of predictors on 1-year milestones [39] shows that our set of perinatal predictors explained much less variance in behavioural development during the second and third year than during the first year, and the lack of variables related to the interaction with adults is a considerable limitation. Thus, children’s milestone development can to a high degree be assumed to be shaped by their learning environment. According to studies of language attainment, the quality of this learning environment has especially been investigated in the home [33–35], and the results emphasize quality of the parent-child interaction [34, 35], maternal responsiveness [36, 37], and appropriate play materials as important predictors [34, 35].

Finally, the possibility of Type I errors is a limitation because we conducted tests on six milestone means (in addition to the Overall mean of milestones) and 22 possible predictors. However, the fact that our findings are generally in agreement with other studies concerning the importance of the predictors suggests that Type I errors are not a substantial problem.

## Conclusion

A total of 16.2% of the variance in the Overall mean of milestones was explained in a final model summarizing the findings from the four domains of predictors: ‘Family background’, ‘pregnancy and delivery’, ‘postnatal factors’, and ‘postnatal growth and development’. The study suggests the following conclusions:

First, there is strong evidence of developmental continuity as the main predictor of behavioural milestones during the second and third years was the Overall 1-year milestone mean, reflecting speed of development during the first year of life.

Second, the sex of the child was of major importance for attainment of milestones during the second and third years with girls showing faster development than boys except for milestones related to Walking and Eating.

Third, parental social status predicted faster attainment of all milestone means except milestones related to Dressing; the strongest association was for milestones related to Language.

Fourth, weight increase during the first year predicted faster attainment of all milestone means except milestones related to Dressing, while foetal growth and growth during the second and third years showed fewer and sometimes inconsistent associations with milestone development.

Fifth, the current study did not analyse potential mediation, but effects of predictors of 1-year milestones such as birth weight and duration of breastfeeding may partly be mediated through later growth and development, in particular the Overall 1-year milestone mean.

Together, such findings may yield an informative screening tool aimed at milestones delay and add valuable knowledge to professionals about how the child’s development can be affected by family background, pregnancy and delivery, postnatal growth and previous development.

Although we were able to explain up to 16.2% of the variance in timing of milestones within specific domains, our findings show that the major part of the variance in milestone attainment depends on variance in environmental or genetic factors other than those incorporated in the four domains in the present study. Thus, more studies are needed to elucidate patterns of these predictors and especially to include more variables related to the actual parent-child interaction.

## Data Availability

The data that support the findings of this study are available from the Steering Committee of the Copenhagen Perinatal Cohort but restrictions apply to the availability of these data, which were used under license for the current study, and so are not publicly available. Data are however available from the authors upon request and with permission of the Steering Committee of the Copenhagen Perinatal Cohort.

## Abbreviation

CPC: Copenhagen Perinatal Cohort
